# The effect of increased plasma potassium on myocardial function; a randomized POTCAST substudy

**DOI:** 10.1007/s10554-023-02914-x

**Published:** 2023-07-20

**Authors:** Ulrik Winsløw, Tharsika Sakthivel, Chaoqun Zheng, Berit Philbert, Michael Vinther, Emil Frandsen, Kasper Iversen, Henning Bundgaard, Christian Jøns, Niels Risum

**Affiliations:** 1 Department of Cardiology, Copenhagen University Hospital, Rigshospitalet, Denmark; 2 Department of Cardiology, Zealand University Hospital, Roskilde, Denmark; 3 Department of Cardiology, Copenhagen University Hospital, Herlev-Gentofte, Denmark; 4 Department of Clinical Medicine, University of Copenhagen, Copenhagen, Denmark

**Keywords:** Potassium, Left ventricular ejection fraction, Global longitudinal strain, Deformation imaging, Mechanical dispersion, Myocardial strain

## Abstract

Plasma potassium (p-K) in the high-normal range has been suggested to reduce risk of cardiovascular arrythmias and mortality through electrophysiological and mechanical effects on the myocardium. In this study, it was to investigated if increasing p-K to high-normal levels improves systolic- and diastolic myocardial function in patients with low-normal to moderately reduced left ventricular ejection fraction (LVEF). The study included 50 patients (mean age 58 years (SD 14), 81% men), with a mean p-K 3.95 mmol/l (SD 0.19), mean LVEF 48% (SD 7), and mean Global Longitudinal Strain (GLS) -14.6% (SD 3.1) patients with LVEF 35–55% from “*Targeted potassium levels to decrease arrhythmia burden in high-risk patients with cardiovascular diseases trial”* (POTCAST). Patients were given standard therapy and randomized (1:1) to an intervention that included guidance on potassium-rich diets, potassium supplements, and mineralocorticoid receptor antagonists targeting high-normal p-K levels (4.5-5.0 mmol/l). Echocardiography was done at baseline and after a mean follow-up of 44 days (SD 18) and the echocardiograms were analyzed for changes in GLS, mechanical dispersion, E/A, e’, and E/e’. At follow-up, mean difference in changes in p-K was 0.52 mmol/l (95%CI 0.35;0.69), P<0.001 in the intervention group compared to controls. GLS was improved with a mean difference in changes of -1.0% (-2;-0.02), P<0.05 and e’ and E/e’ were improved with a mean difference in changes of 0.9 cm/s (0.02;1.7), P = 0.04 and ? 1.5 (-2.9;-0.14), P = 0.03, respectively. Thus, induced increase in p-K to the high-normal range improved indices of systolic and diastolic function in patients with low-normal to moderately reduced LVEF.

**Supplementary Information:**

The online version contains supplementary material available at 10.1007/s10554-023-02914-x.

## Introduction

Low as well as high plasma-potassium (p-K) levels have been shown to be associated with reduced survival [1]. This may in part be related to observed effects of p-K levels on e.g. arrhythmic threshold [2–4], blood pressure [5], and the risk of stroke [6–8] but changes in p-K may also potentially impact the mechanical function of the myocardium.

Myocardial function at low p-K levels has been studied experimentally in dogs (n = 27) and in healthy human volunteers (n = 10) [9, 10]. In these studies, both invasively- and echocardiographically determined indices of systolic- and diastolic function were reduced during potassium depletion. Reduced systolic- and diastolic cardiac function have also been reported in patients with chronically low p-K levels due to primary hyperaldosteronism (n = 62–85) [11–14]. The potential effects of high-normal p-K on myocardial function remain to be explored. Of interest, an observational study of patients with heart failure (n = 6,073) demonstrated that high-normal p-K was associated with a 22% mortality reduction when compared to the normal reference range after less than two years of observation [1]. This is mainly considered to be caused by lower arrhythmia threshold and susceptibility to sudden cardiac death due to a reduction in repolarization reserve [15]. It is, however, unknown whether the association between p-K and outcome is driven by purely electrical changes or in part by mechanical effects on the myocardium.

2D-speckle tracking echocardiography has been demonstrated to be a sensitive and robust method for detection of subtle systolic dysfunction as in preclinical heart failure [16–18]. Furthermore, measurements of the velocity of myocardial motion by tissue Doppler imaging have improved detection of diastolic dysfunction [19]. In this randomized intervention study, we hypothesized that an induced increase in p-K to the high-normal range in patients with low normal to moderately reduced left ventricular ejection fraction (LVEF) is associated with improved systolic myocardial function as assessed by 2D-speckle tracking analysis and with improved diastolic function as measured by conventional clinical methods.

## Materials and methods

### Population

The current substudy was prespecified in the protocol for the *Targeted potassium level to decrease arrhythmia burden in high-risk patients with cardiovascular diseases* (POTCAST) trial (www.clinicaltrials.gov [NCT03833089]). The design of the POTCAST trial has been described previously [20]. In brief, the POTCAST trial is an ongoing randomized clinical trial aiming at enrolling 1,000 patients at a high risk of malignant arrythmia, defined as patients with an implantable cardioverter defibrillator (ICD) implanted as part of routine clinical management for primary or secondary prevention due to inherited or acquired heart diseases. Patients are randomized (1:1) through a concealed computer-generated sequence (project-RedCap.org) [21, 22] to either usual standard of care or usual standard of care with the addition of dietary guidance on increased potassium intake, oral potassium supplements, and mineralocorticoid receptor antagonists (MRA) to increase and maintain p-K between 4.5 mmol/l and 5.0 mmol/l. Inclusion criteria for POTCAST are screening p-K ≤ 4.3 mmol/l and treatment with an ICD. Exclusion criteria are an eGFR < 30 ml/min/1.73 m^2^, pregnancy, seeking pregnancy, or lack of ability to provide informed consent.

For the present study, 50 consecutive patients from the POTCAST trial with LVEF between 35 and 55% were included between June 1, 2020 and May 31, 2021. Patients were excluded from the final analysis if any of the following criteria were present: echocardiographic image quality too poor for 2D-speckle tracking criteria, acute coronary events during follow-up, any changes in heart failure medication during follow-up unrelated to the study, and difference in rhythm at baseline and follow-up echocardiographies.

### Ethics

The study was performed according to the declaration of Helsinki. All patients provided informed consent. The study was approved by regional Danish committee of health research ethics (Regional Videnskabsetisk komité) — Protocol approval no. H-18,044,908 and by the Danish Data Protection Agency — approval no. VD-2018-453.

### Intervention

Patients in the control group continued standard medical treatment. In addition to the standard treatment patients in the intervention group were educated in intake of a potassium rich diet and commenced oral potassium supplement and/or MRA (spironolactone or eplerenone) according to the POTCAST protocol [20]. Because myocardial deformation imaging is sensitive to loading conditions, potassium supplements were chosen as first line intervention to reduce variance in preload and afterload caused by a blood pressure reduction of MRAs. If target p-K levels could not be reached with potassium supplements alone, MRA was used as second line treatment. With close monitoring of renal function and blood pressure, patients were given incremental doses of study medication until target p-K (4.5-5.0 mmol/l) or maximum dosages of potassium supplement (4500 mg ~ 60 mmol) and MRA (50 mg eplerenone or 100 mg spironolactone) were reached.

### Screening and follow-up

Transthoracic echocardiography was performed at enrollment after measurements of p-K^+^, p-Na^+^, p-Mg^2+^, p-Ca^2+^, and p-creatinine and of the blood pressure. Repeated echocardiography was scheduled as soon as possible after either target p-K was met, or maximum dose of study medication was reached. Patients allocated to the control group were scheduled to repeated echocardiography six weeks after baseline due to preliminary results from the POTCAST trial showing that it takes six weeks on average to reach target p-K of 4.5-5.0 mmol/l. P-K and blood pressure were measured immediately after the follow-up echocardiography in both groups. The follow-up echocardiography was scheduled approximately at the same time of day as the baseline echocardiography in order to reduce variance caused by circadian changes in volume status, blood pressure and p-K as well as variation caused by the pharmacokinetics of the patients’ other daily medication.

### Echocardiography

**Fig. 1:**
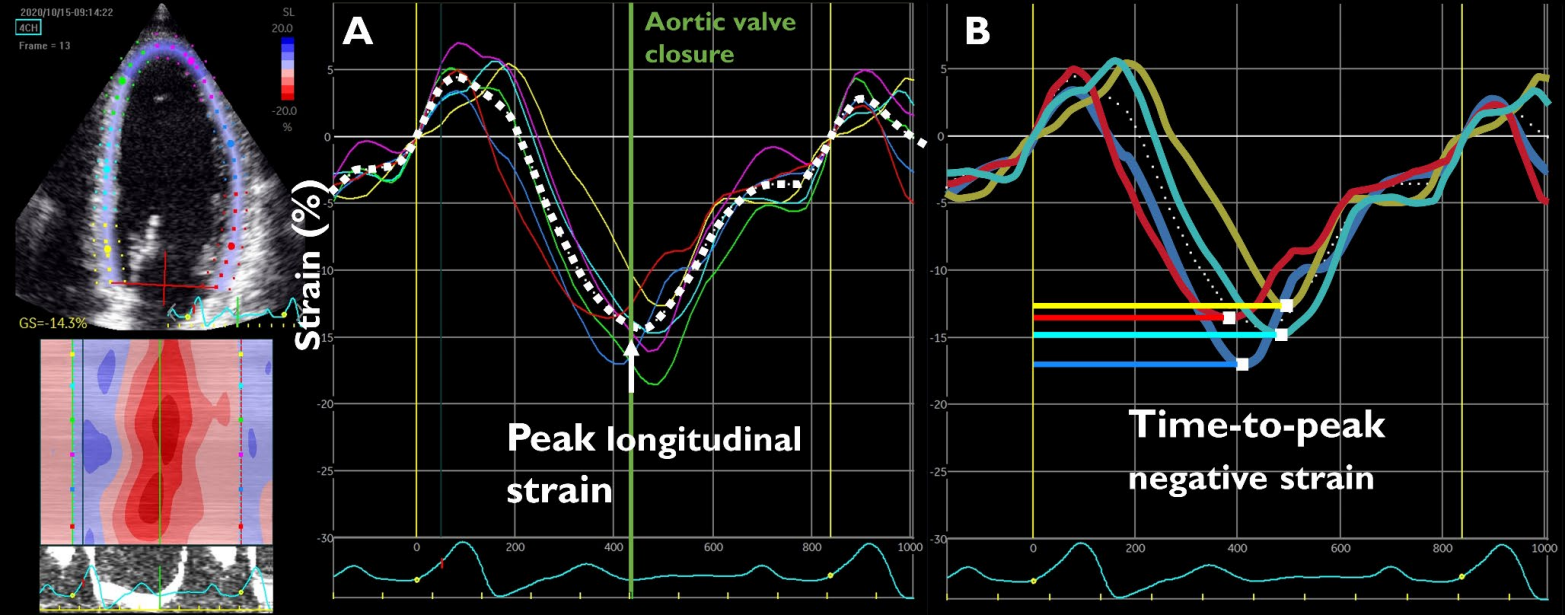
Measurement of Global Longitudinal Strain (A) and Mechanical Dispersion (B) from 2D-Speckle-Tracking software

All echocardiographies were performed by a single investigator using the same ultrasound system (Vivid E9, GE healthcare), the same probe (M5S-D, GE) and the same analyzing software (EchoPac, version 203.66). Cine loops from 3 standard apical views (2-chamber, 4-chamber, and apical long-axis) were recorded using gray-scale harmonic imaging and saved in raw data format. Images were obtained at an acquisition rate of 50 to 90 frames per second. Left ventricular end-diastolic and end-systolic volumes and left ventricular ejection fraction (LVEF) were obtained using Simpson’s biplane method. For 2D-speckle tracking analysis, the inner endocardial borders were traced in the end-systolic frame in images from the three standard apical views. Speckles were tracked frame by-frame throughout the left ventricular wall during the cardiac cycle and basal-, mid-, and apical regions of interest were drawn (Fig. 1). Segments that failed to track were manually adjusted by the operator. Any segments that subsequently failed to track were excluded. GLS was calculated as the mean peak negative longitudinal strain before aortic valve closure of all successfully tracked segments according to an 18-segment model, and mechanical dispersion (MD) was calculated as the standard deviation of time-to-peak negative longitudinal strain in the 12 basal and midventricular segments [23]. Measurements of diastolic function included early diastolic mitral inflow velocity (E), late diastolic mitral inflow velocity (A), E/A ratio, and the average of the septal and lateral early diastolic mitral annular velocity (e’) [24]. Right ventricular function was evaluated by Tricuspid Annular Plane Systolic Excursion (TAPSE) and right ventricular free wall strain (RV FWS). For RV FWS, end-systole was defined at the time of pulmonic valve closure.

Off-line image analyses were independently performed by two investigators blinded to randomization allocation, clinical data and blood test results to reduce the risk of bias.

### Outcomes

The predefined outcomes were differences in changes from baseline to follow-up between the two groups in 2D-speckle tracking derived indices of systolic function: GLS, MD and for assessment of diastolic function: E/A, e´ and E/e´.

### Sensitivity analysis

A subgroup analysis was performed on patients in the intervention group that only received dietary guidance and potassium as a supplement to reach target p-K levels to test if the effects of increasing potassium levels on myocardial function was independent of the effects of MRA on loading conditions.

### Statistics

Values are summarized as mean (standard deviation) or median (interquartile range). Continues data of normal distribution were compared using students t-test. Within-individual comparisons between baseline and follow-up echocardiographies were performed using the paired t-test. Fisher’s Exact test was used to compare categorical variables. Mean difference in changes was calculated as the between group difference in paired estimates to control for random differences in baseline measurements. Thus, the presented results represent absolute changes between study groups. Estimated differences are presented with 95% confidence intervals (95%CI). General linear models were used to assess the correlation between p-K and echocardiographic measurements and presented with R^2^ values obtained from the final models. Interobserver variations was estimated as Single Score Intraclass Correlation. Analyses were done using a standard statistical software program (R version 4.1.0).

### Sample size calculation

To detect a clinically meaningful difference in changes in GLS of 1% with a standard deviation of 1.5 and with a two-tailed α = 0.05 and 1-β = 0.8 in a 1:1 randomization design, 21 patients in each group were needed. A total of 50 patients were included to account for potential dropouts.

## Results

Fifty patients with low normal to moderately reduced LVEF were randomized: 26 patients to the intervention group and 24 to the control group. The participants had a mean age of 58 years (SD 14) and 81% were male. The mean p-K was 3.95 mmol/l (SD 0.19), mean LVEF was 48% (SD 7), and mean GLS was − 14.6% (SD 3.1%). Baseline clinical history, medication, blood test results, and echocardiographic parameters were similar in the two groups (Table 1). Seventeen (36%) patients had ischemic cardiomyopathy, 15 (32%) had dilated cardiomyopathy, and the remaining patients had other heart diseases including arrhythmogenic right ventricular cardiomyopathy (2), hypertrophic cardiomyopathy (2), idiopathic VF (6), and heart failure due to other causes (5).

**Table 1:** Baseline characteristics for patients in the intervention and control group

	Intervention group (n = 25)	Control group (n = 22)
**Age, years**	58 (± 15)	58 (± 13)
**Male sex, n (%)**	12 (80)	20 (91)
**BMI, kg/m** ^**2**^	25.9 (± 4.39)	27.5 (± 5)
**Potassium supp., n(%)**	3 (12)	6 (27)
**MRA treatment, n(%)**	8 (32)	5 (23)
**Beta Blocker treatment, n(%)**	18 (72)	16 (73)
**ACEi/ARB treatment, n(%)**	17 (68)	13 (59)
**Diuretic treatment, n(%)**	9 (36)	9 (41)
**IHD, n(%)**	7 (28)	10 (45)
**DCM, n(%)**	9 (36)	7 (32)
**Afib, n(%)**	2 (8)	2(9)
**Diabetes, n(%)**	1 (4)	2 (9)
**p-K, mmol/l**	3.92 (± 0.19)	3.98 (± 0.2)
**p-Na, mmol/l**	140 (± 2)	141 (± 3)
**p-Mg, mmol/l**	0.85 (± 0.051)	0.85 (± 0.071)
**p-creatinine, µmol/l)**	88 (± 22.2)	82.2 (± 14.7)
**Syst. blood pressure, mmHg**	127 (± 19)	133 (± 15)
**Diast. blood pressure, mmHg**	80.4 (± 13)	81.7 (± 9.8)
**GLS, %**	-14.7 (± 3.12)	-14.5 (± 3.2)
**MD, ms**	52.2 (± 17)	60.6 (± 18.4)
**LVEF, %**	48.7 (± 6.11)	47.5 (± 7.1)
**E, cm/s**	61.1 (± 28.3)	62 (± 24.3)
**A, cm/s**	52.8 (± 19.7)	51.2 (± 12.1)
**E/A**	1.27 (± 0.79)	1.21 (± 0.52)
**e’, cm/s**	7.1 (± 2.23)	7.52 (± 2.42)
**E/e’**	9.49 (± 4.81)	9.01 (± 3.65)
**TAPSE, cm**	2.18 (± 0.38)	2.12 (± 0.55)
**RV FWS, %**	-23.6 (± 5.31)	-21.7 (± 4.96)

Displayed as mean (± SD) or number (%). A: Late diastolic mitral inflow velocity, ACEi: Angiotensin converting enzyme inhibitor, Afib: Atrial fibrillation, ARB: Angiotensin-II receptor blocker, DCM: Dilated cardiomyopathy, E: early diastolic mitral inflow velocity, e’: Early diastolic mitral annular velocity, GLS: Global Longitudinal Strain, IHD: ischemic heart disease, LVEF: Left ventricular ejection fraction, MD: Mechanical dispersion, MRA: Mineralocorticoid receptor antagonist, RV FWS: Right ventricular free wall strain, TAPSE: Tricuspid annular plane systolic excursion

### Follow-up

All patients completed the study and all patients in the intervention group reported full compliance to medication during the study period. Patients in the intervention group were given a mean daily dose of oral potassium supplement of 2,700 mg (~ 36 mmol) and mean dose of MRA of 23 mg. The mean difference in changes in p-K was 0.52 mmol/l (0.35; 0.69), P < 0.001 higher in the intervention as compared to the control group.

**Fig. 2:**
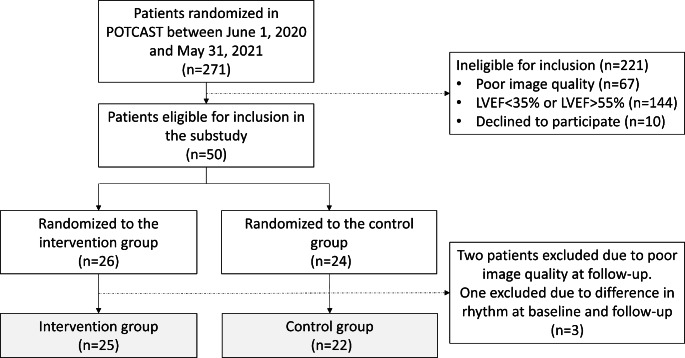
Flowchart showing patient randomization in the POTCAST trial from June 1st 2020 until May 31st 2021 and derivation of study population in the current substudy

The follow-up echocardiography was performed after a mean of 44 days (SD 18) after baseline, 47 days (SD 17) in the intervention group and 40 days (SD 18) in the control group (P = 0.22). Three patients were excluded from the final analysis: two due to poor image quality and one due to differences in rhythm at baseline and follow-up (sinus rhythm and atrial fibrillation) — Fig. 2. Table 2 shows clinical characteristics and echocardiographic parameters at follow-up between the two groups. Notably, there were no statistically significant differences in changes in systolic blood pressure, diastolic blood pressure, or heart rate between the groups.

**Table 2:** Changes in clinical characteristics and echocardiographic parameters between the intervention group (n = 25) and the control group (n = 22) from baseline to follow-up. Difference in changes is calculated as change in the intervention group relative to the control group

	Intervention (Follow-up)	Change from baseline	Control (Follow-up)	Change from baseline	Diff. in changes from baseline	P-Value
**P-K, mmol/l**	4.51 (± 0.36)	0.59 (± 0.34)	4.05 (± 0.17)	0.068 (± 0.25)	0.52 (0.35; 0.69)	< 0.001*
**Systolic bp, mmHg**	123 (± 11.2)	-3.92 (± 14.4)	129 (± 14.9)	-3.68 (± 10.6)	-0.3 (-7.6; 7.2)	0.95
**Diastolic bp, mmHg**	78.2 (± 9.3)	-2.2 (± 12.3)	79.9 (± 10.3)	-1.82 (± 12)	-0.4 (-7.5; 6.8)	0.91
**HR, (bpm)**	63.3 (± 8.9)	0.85 (± 7.22)	61.3 (± 10.9)	0.061 (± 5.94)	0.8 (-3.1; 4.7)	0.68
**Systolic function**						
**GLS, %**	-15.8 (± 3.5)	-1.1 (± 1.39)	-14.6 (± 3.3)	-0.068 (± 1.94)	-1.0 (-2.0; -0.02)	< 0.05*
**LVEF, %**	49 (± 7.17)	0.28 (± 3.55)	46.3 (± 8.21)	-1.23 (± 5.52)	1.5 (-1.3; 4.3)	0.28
**MD, ms**	48.7 (± 19.7)	-3.55 (± 11.9)	60.8 (± 18.4)	0.146 (± 15)	-3.7 (-11.8; 4.36)	0.36
**TAPSE, cm**	2.11 (± 0.36)	-0.072 (± 0.35)	1.98 (± 0.53)	-0.15 (± 0.35)	0.07 (-0.13; 0.28)	0.29
**RV FWS, %**	-24.5 (± 5.77)	-0.88 (± 4.36)	-22.4 (± 5.22)	-0.76 (± 3.31)	-0.12 (-2.58; 2.34)	0.92
**Diastolic function**						
**E, cm/s**	56.6 (± 24.5)	-4.48 (± 10.4)	60.5 (± 23.6)	-1.41 (± 9.42)	-3.07 (-8.89; 2.75)	0.29
**A, cm/s**	50.1 (± 15.3)	-3.43 (± 13.6)	49.4 (± 11.9)	-1.7 (± 4.35)	-1.73 (-7.89; 4.43)	0.57
**E/A**	1.2 (± 0.734)	-0.0013 (± 0.21)	1.25 (± 0.59)	0.040 (± 0.24)	-0.041 (-0.18; 0.10)	0.56
**e’, cm/s**	7.68 (± 2.69)	0.58 (± 1.44)	7.25 (± 2.36)	-0.27 (± 1.39)	0.85 (0.02; 1.68)	0.04*
**E/e’**	7.97 (± 2.85)	-1.52 (± 2.81)	9.03 (± 3.87)	0.023 (± 1.89)	-1.54 (-2.94; -0.14)	0.03*

P-value for difference in mean change in the intervention group and the control group

Displayed as mean (± SD) or number (%). A: Late diastolic mitral inflow velocity, E: early diastolic mitral inflow velocity, e’: Early diastolic

mitral annular velocity, HR: Heart rate, LVEF: Left ventricular ejection fraction, GLS: Global Longitudinal Strain, MD: Mechanical dispersion, RV FWS: Right ventricular free wall strain, TAPSE: Tricuspid annular plane systolic excursion

### Systolic function

**Fig. 3:**
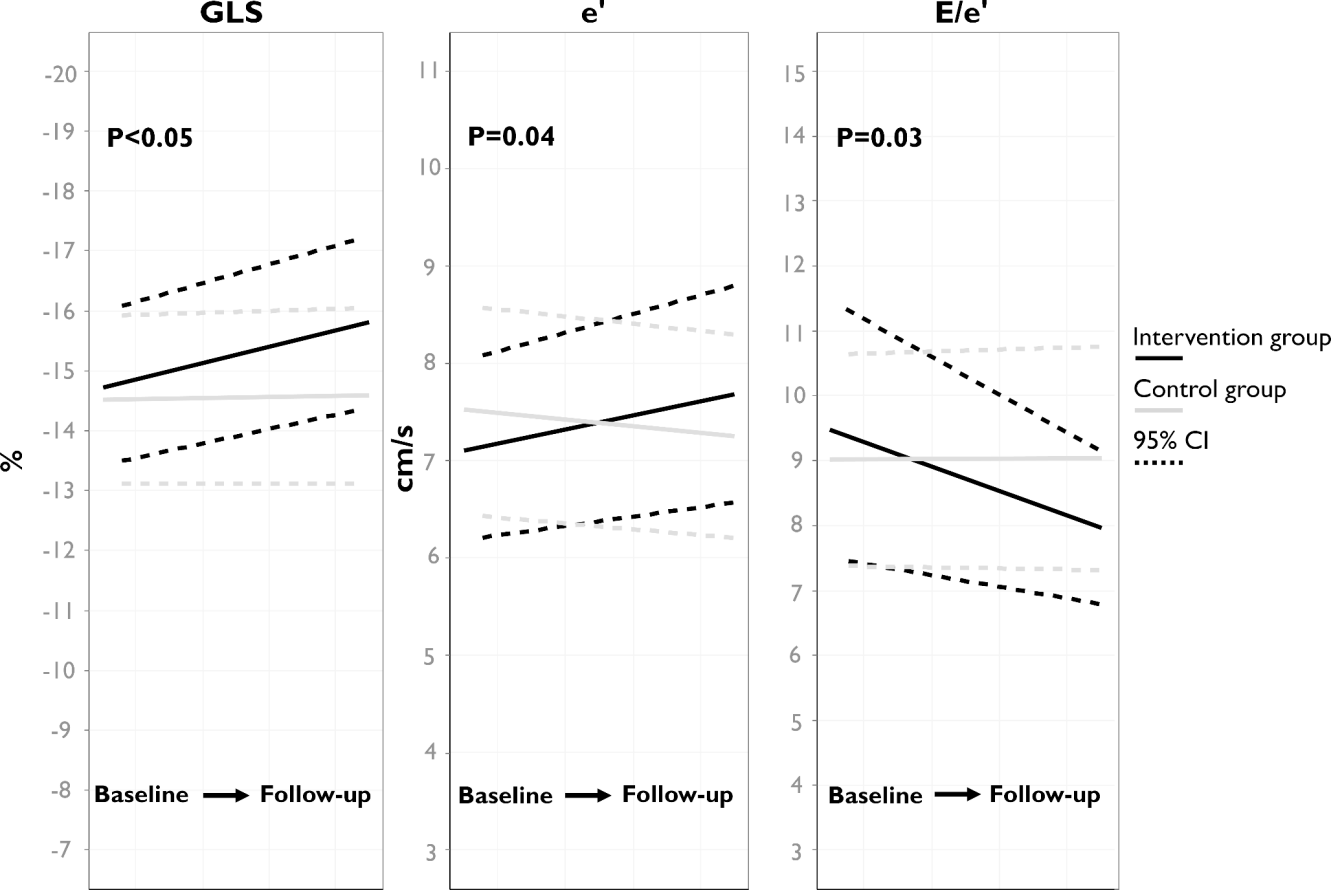
Mean GLS, e’, E/e’ along with 95% Confidence Interval in the intervention- and control group at baseline and follow-up

At follow-up, the intervention group had improved myocardial longitudinal contraction measured by GLS with a mean difference in changes of -1.0% (-2.0; -0.02), P < 0.05 compared to controls (Fig. 3). No significant difference in changes in contractile heterogeneity as determined by mechanical dispersion was found in the intervention group compared to controls (-3.7 ms (-3.5;0.1), P = 0.36). No significant difference in changes in LVEF or RV systolic measures (TAPSE and RV FWS) were observed between groups (Table 2).

### Diastolic function

The intervention group had improved diastolic function as measured by e’ with mean difference in changes of 0.9 cm/s (0.02; 1.7), P = 0.04 and improvement in E/e’ with mean difference in changes of -1.5 (-2.9; -0.1), P = 0.03 compared to controls. No significant difference in changes was found between groups for E, A, or E/A.

### Relation between p-K and mechanical function

In multiple linear regression models, no correlation was found between p-K and any of the echocardiographic parameters that were improved with the intervention. GLS (ß=-0.9, P = 0.22, R^2^ = 0.04), e’ (-0.068, P = 0.91, R^2^ = 0.01) or E/e’ (with ß=-0.91, P = 0.37, R^2^ = 0.07). Models were adjusted for baseline values of p-K to account for regression to the mean. No association was found between GLS and change in systolic blood pressure (ß=0.02, P = 0.51, R^2^ = 0.01), change in diastolic blood pressure (ß=0.04, P = 0.17, R^2^ = 0.05), or change in heart rate (ß=-0.02, P = 0.53, R^2^ = 0.01) in linear models.

### Sensitivity analysis

Patients in the intervention group only receiving potassium as a supplement (n = 15) had similar baseline characteristics as the control group (Supplementary Table S1). No interaction was found between MRA treatment and the effect of the intervention on any of the parameters investigated (Supplementary Table S2). In the intervention subgroup mean differences in changes in p-K was 0.49 (0.31; 0.68), P < 0.001 compared to controls. The improvement in systolic- and diastolic function was robust in subgroups. In the intervention group mean difference in change in GLS was − 1.2 (-2.3; -0.06), P = 0.04) compared to controls while mean difference in changes in e’ was 0.9 cm/s (-0.05; 1.9), P = 0.06 and in mean difference in changes in E/e’ was − 1.3 (-2.8; 0.2), P = 0.1 compared to controls.

Interobserver variability analysis showed close correlation between the two independent observers. Intraclass correlation coefficient: ICC = 0.72, P = 0.006.

### Safety

No patient in either group was hospitalized due to electrolyte disturbance or renal failure between baseline and follow-up.

## Discussion

This study was designed to determine whether increased potassium levels influence systolic and diastolic myocardial performance. It is the first randomized intervention study to indicate that targeting high-normal p-K levels using dietary guidance, oral potassium supplements and MRAs improves echocardiographic indices of myocardial systolic and diastolic function. No difference in changes was found in contractile heterogeneity measured by MD between groups.

The findings of the present study are in line with previous experimental and observational studies investigating myocardial mechanical function in relation to potassium depletion. Induced potassium depletion in a canine-model (n = 27) has been associated with impaired systolic and diastolic responses to stress tests with epinephrine and increased preload [9]. Potassium depletion of healthy adults (n = 10) to a p-K < 3.5 mmol/l caused an impairment in echocardiographic indices of diastolic function [10]. In a recent study of patients (n = 67) with chronic potassium depletion due to primary hyperaldosteronism impairment in GLS was demonstrated [12]. In these studies, subjects were hypokalemic which diverts from the current study in which an increase in p-K from a normokalemic to high-normal level was investigated.

In exploratory analysis of a subset of 131 patients from the TOPCAT trial, there was a trend towards improvement of Longitudinal Strain in patients treated with spironolactone (1.1% [-0.2;-2.4], P = 0.09) [18]. Additionally, an association between spironolactone and improvement in diastolic function by e’ and E/e’ has been demonstrated in another randomized trial of patients with heart failure with preserved ejection fraction. E’ was increased by 0.4 cm/s (0.1–0.6), P = 0.002 and E/e’ was decreased by 1.5 (-2.0;-0.9), P < 0.001 in the spironolactone arm [25]. These findings are consistent with those from the current study. MRAs have effects on the renin-angiotensin-aldosterone-system, on prevention of adverse myocardial remodeling and fibrosis, collagen metabolism which could affect myocardial function independent of blood pressure [26]. Of note however, is that the sensitivity analysis demonstrated that the findings in the present study were similar for a subgroup of patients in the intervention group that were only treated with potassium supplements. Furthermore, no difference in systemic blood pressure was found between groups. This indicates that the effect of the intervention was primarily related to the increased p-K levels and not to other effects of MRA.

### The effects of potassium on cardiovascular risk

The protective effects of high potassium intake on cardiovascular outcomes have been reported in several studies. The potassium-induced lowering effect on blood pressure in hypertensive patients and reduction in the risk of strokes is particularly well documented [5, 6]. Randomized trials have shown that increased potassium intake and reduced sodium intake are associated with lower rates of major adverse cardiovascular events and death from all causes [7, 27]. Additionally, multiple observational studies have shown that higher potassium levels reduce the risk of both supraventricular and ventricular tachyarrhythmias [2–4]. Yet, current clinical guidelines only make specific recommendations on sodium intake but potassium intake in patients with cardiovascular diseases is not addressed. The present study adds that increased potassium intake also seems to increase mechanical myocardial function and might in part explain the positive effect seen on cardiovascular risk.

To our knowledge this is the first study to investigate myocardial function after actively increasing potassium levels in a randomized controlled setting. The study showed an improvement in GLS which has been repeatedly reported to be a strong predictor of arrhythmias [28, 29], and improvement in e’ and E/e’ which have been shown to be associated with all-cause mortality and cardiovascular hospitalizations [30, 31]. Thus, the echocardiographic parameters that were improved in the current study links mechanical left ventricular function to electrophysiological events. E/e’ has also been associated with left ventricular filling pressure, left atrial pressure and diastolic function. These parameters are related to many variables which could be affected indirectly by an increase in potassium intake such as loading conditions. This complicates interpretation of the mechanism behind the association between the intervention and E/e’ presented in this study. The effect sizes of the potassium-increasing intervention on GLS, e’ and E/e’ were small. Still the observed improvements in mechanical function may relate to the well-known protective effects of potassium on cardiovascular risk. A 24% reduction in heart failure events and death has previously been reported that for every 1% improvement in GLS [32]. The effect of potassium-increasing intervention on arrhythmias and other cardiovascular outcomes observed in earlier studies may therefore not be purely electrical.

### Possible mechanism for improved myocardial function with higher potassium levels

Changes in potassium concentrations in- and around the cardiomyocyte affect several aspects of myocardial electrophysiology which could result in altered mechanical function. P-K determines the resting membrane potential of the myocyte and significantly impacts depolarization velocity. Additionally, cardiac repolarization is mainly driven by an outward potassium flux and a low p-K lengthens the repolarization time and increases QT dispersion and active relaxation of the myocardium as well as the action potential- and refractory period durations [15]. Thus, the effect of increased p-K on systolic contraction amplitude and diastolic relaxation might be the result of these mechanisms, but further studies are needed.

The effects of variations in potassium levels are likely to be more pronounced in patients with heart failure as the remodeling of the myocardium during deterioration of left ventricular function and increased hemodynamic load, even in early stages of heart failure, includes downregulation of potassium ion channels as well as of myocardial Na,K- ATPase concentration [33], responsible for cardiac repolarization, making them sensitive to low potassium conditions [34, 35]. The improvement in myocardial function found in the current study could not be directly correlated to p-K, this indicates that at least parts of the effects are independent of the extracellular potassium levels. Recent findings in the POTCAST trial have demonstrated that increased potassium intake and use of MRAs not only increases p-K but also intracellular potassium (Winsløw, unpublished data — under review, 2023). Thus, the results in the current study may reflect direct- or indirect effects of increased intracellular potassium. A pharmacologically induced increase in intracellular potassium could improve contractility as well as impulse generation and -propagation by lowering intracellular sodium which is a hallmark of heart failure [36]. Lastly, potassium has been demonstrated to reduce oxidative stress which can induce abnormal left ventricular relaxation even in early stages of heart failure [37, 38].

Patients in the present study were increased from mid-normal to high-normal levels of p-K and there could be a greater improvement by increasing patients from low or even hypokalemic levels to high-normal levels. These considerations are left for future studies. Studies with hard endpoints are needed to elucidate if the present findings improve clinical patient outcomes.

### Strength and limitations

The randomized controlled design strengthens the causal inference of the findings. The present study was open labelled and therefore patients in the control group could have been motivated to increase their potassium intake independent of the trial. This is not likely to be an important factor as p-K levels in the control group were unchanged from baseline to follow-up. The study was limited by a low sample size and larger trials are needed to confirm the findings. Further, the study was designed as an all-comer trial of patients with low-normal- to moderately reduced left ventricular ejection fraction. Thus, the cohort is heterogeneous which could affect the generalizability of the results. The primary analysis is not conclusive on whether the effects seen with the intervention on myocardial function is caused by changes in potassium homeostasis per se or the effects of MRAs. However, the sensitivity analysis of patients only receiving potassium supplements showing similar results strengthens confidence in the main hypothesis.

## Conclusion

Targeting p-K between 4.5 and 5.0 mmol/l with dietary guidance on a potassium rich diet, oral potassium supplements and MRAs improved indices of systolic and diastolic left ventricular function in patients with low-normal to moderately reduced LVEF. These findings may in part explain previously reported beneficial effects of increased potassium intake and prove to be of clinical importance.

### Electronic supplementary material

Below is the link to the electronic supplementary material.

Supplementary Material 1

## Data Availability

The data is available from the corresponding author on reasonable request in accordance with the Danish Data Protection Act and the General Data Protection Regulation and after the approval by the steering committee of the POTCAST trial.

## Declarations

### Transparency statement

The authors had full access to and take full responsibility for the integrity of the data. All authors have read and agree to the manuscript as written.
