# Supplementary material for: The effect of increased plasma potassium on myocardial function; a randomized POTCAST substudy
Source: Int J Cardiovasc Imaging. 2023 Jul 20;39(11):2097–106. doi: 10.1007/s10554-023-02914-x (PMC10673982; doi:10.1007/s10554-023-02914-x)
Supplement: Supplementary file 1 — Supplementary Material 1 [file 10554_2023_2914_MOESM1_ESM.docx]

**Supplementary Appendix**

The effect of increased plasma potassium on myocardial function; a randomized POTCAST substudy

*Subgroup analysis of patients in the intervention group treated with potassium supplement alone*
A subgroup analysis was performed on 15 patients in the intervention group that were only treated with potassium supplements to reach target p-K levels. This was done to test if the effects of increasing potassium levels on myocardial function is independent of the effects of MRA on loading conditions, adverse remodeling, myocardial fibrosis, and collagen metabolism. Baseline characteristics in this subgroup and the control group were similar (Supplementary Table S1). However, blood pressure was significantly lower in comparison to the control group at baseline (systolic blood pressure: 119 (±14.2) vs 133 (±15.4), P=0.008, diastolic blood pressure: 74.3 (±10) vs 81.7 (±9.8), P=0.032).

No interaction was found between MRA treatment and the effect of the intervention on any of the parameters investigated (Supplementary Table S2).

**Supplementary Table S1:** Baseline characteristics for patients in the intervention group that only received potassium as a supplement (n=15) and the control group (n=22).

|  | **Intervention group (n=15)** | **Control group (n=22)** | **P-value** |
| --- | --- | --- | --- |
| **Age, years** | 59.2 (±11.7) | 57.5 (±12.8) | 0.67 |
| **Male gender, n(%)** | 18 (72) | 20 (91) | 0.38 |
| **BMI, kg/m^2^** | 25.5 (±4.26) | 27.5 (±5) | 0.2 |
| **Potassium supp, n(%)** | 2 (13) | 6 (27) | 0.43 |
| **MRA treatment, n(%)** | 6 (40) | 5 (23) | 0.30 |
| **Beta Blocker treatment, n(%)** | 10 (67) | 16 (73) | 0.73 |
| **ACEi/ARB treatment, n(%)** | 11 (73) | 13 (59) | 0.49 |
| **Diuretic treatment, n(%)** | 5 (33) | 9 (41) | 0.74 |
| **IHD, n(%)** | 4 (27) | 10 (45) | 0.31 |
| **DCM, n(%)** | 6 (40) | 7 (32) | 0.73 |
| **Afib, n(%)** | 2 (13) | 2 (9) | 1.00 |
| **Diabetes, n(%)** | 1 (7) | 2 (9) | 1.00 |
| **p-K, mmol/l** | 3.96 (±0.21) | 3.98 (±0.2) | 0.31 |
| **p-Na, mmol/l** | 140 (±2) | 141 (±3) | 0.71 |
| **p-Mg, mmol/l** | 0.84 (±0.045) | 0.85 (±0.071) | 0.90 |
| **p-creatinine, µmol/l)** | 93.1 (±25.3) | 82.2 (±14.7) | 0.29 |
| **Syst. blood pressure, mmHg** | 119 (±14) | 133 (±15) | 0.01 |
| **Diast. blood pressure, mmHg** | 74.3 (±10) | 81.7 (±9.8) | 0.03 |
| **GLS, %** | -14 (±3.47) | -14.5 (±3.17) | 0.64 |
| **MD, ms** | 55.1 (±17.2) | 60.6 (±18.4) | 0.11 |
| **LVEF, %** | 47.5 (±7.06) | 47.5 (±7.1) | 0.97 |
| **E, cm/s** | 65.8 (±34.6) | 62 (±24.3) | 0.71 |
| **A, cm/s** | 49.5 (±22.1) | 51.2 (±12.1) | 0.8 |
| **E/A** | 1.43 (±0.925) | 1.21 (±0.523) | 0.42 |
| **e', cm/s** | 7.2 (±2.24) | 7.52 (±2.42) | 0.68 |
| **E/e'** | 9.8 (±4.99) | 9.01 (±3.65) | 0.61 |
| **TAPSE, cm** | 2.15 (±0.358) | 2.12 (±0.552) | 0.87 |
| **RV FWS, %** | -23.5 (±4.78) | -21.7 (±4.96) | 0.25 |

Displayed as mean (± SD) or number (%). A: Late diastolic mitral inflow velocity, ACEi: Angiotensin converting enzyme inhibitor, Afib: Atrial fibrillation, ARB: Angiotensin-II receptor blocker, DCM: Dilated cardiomyopathy, E: early diastolic mitral inflow velocity, e’: Early diastolic mitral annular velocity, GLS: Global Longitudinal Strain, IHD: ischemic heart disease, LVEF: Left ventricular ejection fraction, MD: Mechanical dispersion, MRA: Mineralocorticoid receptor antagonist, RV FWS: Right ventricular free wall strain, TAPSE: Tricuspid annular plane systolic excursion.

**Supplementary Table 2:** Changes in clinical characteristics and echocardiographic parameters between the intervention group that only received potassium as a supplement (n=15) and the control group (n=22) from baseline to follow-up. Difference in changes is calculated as change in the intervention group relative to the control group.

|  | **Intervention (Follow-up)** | **Change from baseline** | **Control (Follow-up)** | **Change from baseline** | **Diff. in changes from baseline** | **P-Value** |  |
| --- | --- | --- | --- | --- | --- | --- | --- |
| **P-K, mmol/l** | 4.52 (±0.326) | 0.56 (±0.28) | 4.05 (±0.171) | 0.068 (±0.25) | 0.49 (0.31; 0.68) | <0.001* | |
| **Systolic bp, mmHg** | 121 (±12.4) | 1.67 (±8.18) | 129 (±14.9) | -3.68 (±10.6) | 5.35 (-0.944; 11.6) | 0.093 | |
| **Diastolic bp, mmHg** | 76.5 (±9.02) | 2.27 (±10.1) | 79.9 (±10.3) | -1.82 (±12) | 4.08 (-3.35; 11.5) | 0.27 | |
| **HR, (bpm)** | 64.5 (±9.17) | 2.73 (±7.42) | 61.3 (±10.9) | 0.061 (±5.94) | 2.67 (-2.05; 7.39) | 0.26 | |
| **Systolic** |  |  |  |  |  |  | |
| **GLS, %** | -15.6 (±3.69) | -1.26 (±1.44) | -14.6 (±3.31) | -0.068 (±1.94) | -1.42 (-2.46; -0.373) | 0.01* | |
| **Diastolic** |  |  |  |  |  |  | |
| **e', cm/s** | 7.87 (±2.78) | 0.67 (±1.48) | 7.25 (±2.36) | -0.273 (±1.39) | 0.93 (-0.051; 1.93) | 0.06 | |
| **E/e'** | 8.54 (±3.26) | -1.26 (±2.48) | 9.03 (±3.87) | 0.023 (±1.89) | -1.28 (-2.84; 0.28) | 0.1 | |

P-value for difference in mean change in the intervention group and the control group.
Displayed as mean (± SD) or number (%). E: early diastolic mitral inflow velocity, e’: Early diastolic

mitral annular velocity, HR: Heart rate, LVEF: Left ventricular ejection fraction, GLS: Global Longitudinal Strain.
